# Proving of Vaccinium

**Published:** 1887-05

**Authors:** S. Swan

**Affiliations:** New York


### PROVING OF VACCININUM.

S. Swan, M. D., New York.

1886, October 19th, Mrs. G. A. took one dose of DM (Swan), and a second dose on October 22d. On October 29th, in morning, was taken with aching pains in back, worse in lumbar region, extending round waist. Tired all over, with stretching, gaping feeling; unnatural fatigue. Full feeling in head, with running at nose; severe headache in vertex.

On morning of 29th and afternoon of 31st slight coldness, followed by fever; ditto between five and six P. M., November 2d. Her rheumatic pains in wrists and hands have gone since taking the medicine.

*Vaccininum* in potency also removed a chronic catarrh.

